# The genetics of obesity - Pathogenetic, clinical and diagnostic aspects

**DOI:** 10.34763/devperiodmed.20172103.186202

**Published:** 2017-10-28

**Authors:** Artur Barczyk, Anna Kutkowska-Kaźmierczak, Jennifer Castañeda, Ewa Obersztyn

**Affiliations:** 1 Zakład Genetyki Medycznej, Instytut Matki i Dziecka, Warszawa, Polska

**Keywords:** otyłość, genetyka, otyłość monogenowa, otyłość zespołowa, otyłość wieloczynnikowa, otyłość u człowieka, obesity, genetics, monogenic obesity, syndromic obesity, multifactorial obesity, human obesity

## Abstract

Due to its prevalence and its health-related, economic and social consequences, childhood and adult obesity is a complex, medical and civilizational problem, which has been on the increase in the last decade. The results of multi-center investigations reveal that genetic factors play an essential role in the etiopathogenesis of obesity, particularly in the case of extreme cases with very early onset. The Body Mass Index (BMI) is one of the most frequently used indicators of obesity and shows a strong genetic component with a 40-70% degree of heritability. The three types of genetically conditioned obesity are: (1) isolated (nonsyndromic) monogenic obesity, (2) syndromic monogenic obesity associated with dysmorphic features and/or congenital defects, caused by mutations in specific gene(s), (3) chromosomal aberrations, including submicroscopic changes. The most prevalent common (complex) obesity is linked to the presence of various changes in different genomic loci, which are subject to interactions and modifications by environmental (ethnic, dietary, lifestyle, bacterial flora, oxidative stress), as well as epigenetic (i.e., associated with DNA methylation, histone modification) and epistatic (gene-gene interaction) factors. Recent investigations using the modern methods of genome-wide association studies (GWAS), bioinformatics and proteomics, have made it possible to elucidate 8 key genes among the 97 genes most likely to play significant roles in the metabolic effects of obesity. The results of investigations on the pathogenesis of complex obesity do not as yet clarify the potential pathogenic significance of these genomic changes in humans. This article discusses the neuro-endocrinological regulation of the sensation of hunger and thirst, the clinical consequences of mutations in genes associated with the melanocortin pathway, and the features of the most common obesity syndromes, including syndromes conditioned by genomic imprinting. A diagnostic algorithm for cases of suspected syndromic obesity is proposed.

## Streszczenie

Otyłość wśród dzieci i dorosłych stanowi złożony, wzrastający w ciągu ostatniej dekady problem medyczny i społeczny zarówno ze względu na jej rozpowszechnienie oraz skutki zdrowotne i ekonomiczne. Jak wskazują wyniki wieloośrodkowych badań, otyłość może być wynikiem interakcji czynników genetycznych i środowiskowych w etiopatogenezie otyłości. Szczególnie w przypadkach tzw. ekstremalnych może być jednym z objawów zespołu genetycznie uwarunkowanego. Wyszczególnia się trzy typy genetycznie uwarunkowanej otyłości: postaci izolowane (niesyndromiczne) uwarunkowane jednogenowo, postaci otyłości monogenowej zespołowej współistniejącej z cechami dysmorfii i/lub wadami wrodzonymi, uwarunkowanej mutacjami określonego genu (ów), aberracjami chromosomowymi, w tym zmianami submikroskopowymi. Najczęściej występująca tzw. otyłość powszechna (kompleksowa) uwarunkowana jest obecnością licznych zmian zlokalizowanych w różnych miejscach genomu, które podlegają interakcjom i modyfikacjom przez czynniki środowiskowe (etniczne, dieta, tryb życia, flora bakteryjna, stres oksydacyjny) i epigenetyczne (np. związane z metylacją DNA, modyfikacja histonów) oraz epistatyczne (oddziaływanie międzygenowe). Odkrycia ostatnich lat dokonane dzięki rozwojowi najnowszych technik badawczych genomu człowieka (GWAS – Genome – WideAssociationStudies), bioinformatyki, proteomiki umożliwiły wyłonienie około 8 kluczowych spośród 97 genów, które prawdopodobnie odgrywają ważną rolę w metabolizmie osób otyłych. Wyniki dotychczasowych badań nad patogenezą otyłości kompleksowej nie wyjaśniają potencjalnego patogennego znaczenia wykrytych zmian w genomie u człowieka. Przedstawiono problematykę neuroendokrynnej regulacji uczucia głodu i sytości oraz skutki kliniczne mutacji genów związanych ze szlakiem melanokortynowym oraz charakterystykę częściej występujących zespołów otyłości syndromicznej, w tym zespołów uwarunkowanych mechanizmem „genomic imprinting”. Zaproponowano algorytm postępowania diagnostycznego w przypadku podejrzenia zespołowych postaci otyłości.

## WSTĘP

Otyłość i nadmierna masa ciała stanowią poważny problem zdrowotny w skali całej populacji i zwykle jest wynikiem nadmiernego gromadzenia tkanki tłuszczowej w organizmie. Powszechnie stosowaną miarą stosowaną dla osób dorosłych jest wskaźnik masy ciała (Body Mass Index, BMI). Nadwaga jest definiowana jako przekroczenie wartości BMI 25, a otyłość 30. W związku ze znaczną zmiennością średnich wartości BMI w okresie rozwojowym definicję nadwagi i otyłości u dzieci do 18 roku życia opiera się obecnie na wartościach centylowych masy ciała (>85 i >95 w USA, >90 i >97 w Europie) [1].

Od końca lat 80 XX wieku obserwuje się stałą tendencję do wzrostu wartości BMI u osób dorosłych o 0,4-0,5 w ciągu dekady [2]. Rośnie odsetek osób z nadwagą i otyłością, w USA w 2000 roku odsetek dorosłych z nadwagą lub otyłością przekroczył odpowiednio 61% i 26%. Trend ten jest także wyraźny w odniesieniu do dzieci i młodzieży. W ciągu ostatnich 3 dekad odsetek otyłych dzieci w wieku 2-5 oraz 12-19 lat potroił się, a czterokrotnie zwiększył się w przedziale wiekowym 6-11 lat; obecnie w USA około 1/3 dzieci i młodzieży ma nadmierną masę ciała [3]. Otyłość w wieku dziecięcym oznacza wysokie ryzyko otyłości także w wieku dorosłym [4], co z kolei niesie liczne zagrożenia dla zdrowia, w postaci zwiększonego prawdopodobieństwa m.in. cukrzycy, choroby wieńcowej, udarów mózgowych, problemów ortopedycznych, obturacyjnego bezdechu sennego, niektórych nowotworów i wczesnego zgonu [5]. Szacunki przewidują, że w przypadku utrzymywania się tego trendu w 2030 roku w całej populacji światowej będzie 38% osób z nadwagą i 20% z otyłością. Zgodnie z danymi World Health Organisation (WHO) około 2,8 milionów ludzi rocznie na całym świecie umiera z powodu otyłości i jej powikłań

Podstawowych przyczyn tego stanu upatruje się w zmianach stylu życia, zwłaszcza coraz łatwiejszej dostępności do żywności wysokoenergetycznej oraz zmniejszającej się aktywności fizycznej w ostatnim półwieczu, a także czynników hormonalnych związanych z przemianą i wydatkowaniem energii podlegającej ośrodkowej regulacji osi przysadka-podwzgórze oraz wzajemnego oddziaływania różnych czynników, w tym czynników genetycznych. W latach 90 tych zwróciło uwagę badaczy rodzinne występowanie otyłości. W rodzinach, w których oboje rodzice są otyli, ryzyko otyłości u dziecka jest 10 krotnie większe od populacyjnego [6]. Zastosowanie klasycznych metod badania bliźniąt monozygotycznych i dizygotycznych wychowywanych razem i osobno, dowiodło, iż czynniki genetyczne odgrywają istotną rolę w etiopatogenezie otyłości, czego wyrazem jest odziedziczalność BMI na poziomie od 40 - 70% [7, 8]. Badania w rodzinach adopcyjnych niespodziewanie wykazały silniejszy wpływ BMI rodziców biologicznych na występowanie otyłości u dzieci, niż BMI rodziców adopcyjnych [9]. Te obserwacje wskazują na istotną rolę uwarunkowań genetycznych jako przyczyny otyłości, szczególnie w przypadkach tzw. ekstremalnych otyłości oraz otyłości z początkiem na bardzo wczesnych etapach rozwoju (okres niemowlęcy, wczesne dzieciństwo). Zapoczątkowane zsekwencjonowaniem genomu człowieka w 2003 roku (Human Genome Project) badania z zastosowaniem nowoczesnych metod biologii molekularnej (wysokoprzepustowe sekwencjonowanie następnej generacji – Genome Wide Association Studies – GWAS) oraz bioinformatyki i statystyki, umożliwiły poznanie wielu genów/miejsc w genomie, które mogą mieć potencjalne znaczenie w rozwoju otyłości u człowieka.

Zgodnie ze stanem współczesnej wiedzy otyłość jest chorobą złożoną, charakteryzującą się zróżnicowaną ekspresją kliniczną oraz heterogenną, etiologią.

Obecnie najczęściej w praktyce klinicznej wyróżnia się trzy typy otyłości:

otyłość jednogenowa (monogenic obesity) – tzw. otyłość izolowana uwarunkowana mutacjami pojedynczych genów.
otyłość syndromiczna, będąca jednym z objawów zespołu genetycznie uwarunkowanego
  − zespoły dysmorficzne uwarunkowane monogenowo (ze współistnieniem wad wrodzonych, i /lub / wadami niepełnosprawnością intelektualną,
  − zespoły aberracji chromosomowych,
  − zespoły mikrodelecji/mikroduplikacji (choroby genomowe), w tym choroby uwarunkowane rodzicielskim piętnowaniem genomowym.
3 otyłość powszechna (common obesity) występującą najczęściej, uwarunkowaną poligenowo, wieloczynnikowo.

## Otyłość Izolowana, Jednogenowa

Znaczący postęp w badaniach nad genetyką otyłości przyniosła ostatnia dekada XX wieku, dzięki wcześniejszym obserwacjom zebranym w badaniach doświadczalnych na modelu zwierzęcym, mających na celu poznanie złożonego mechanizmu ośrodkowej regulacji wydzielania hormonów, białek i neuroprzekaźników zapewniających utrzymanie homeostazy związanej z poborem i wydatkowaniem energii oraz ich wpływu na uczucie głodu i sytości. Pierwsze odkrycia dotyczyły lokalizacji podwzgórzowego układu regulacji głodu i sytości, co było możliwe dzięki eksperymentalnym uszkodzeniom podwzgórza u szczurów. Badania doświadczalne na modelach mysich doprowadziły do odkrycia u skrajnie otyłych myszy ob/ ob i db/db mutacji genu leptyny i jej receptora, co miało fundamentalne znaczenie dla lepszego poznania neurohormonalnych mechanizmów modulujących nadmierne odkładanie tkanki tłuszczowej, a w szczególności roli podwzgórza w kontroli sytości i głodu [10].

Podstawowym ośrodkiem regulacji apetytu, który nosi nazwę szlaku melanokortynowego, jest jądro łukowate w podwzgórzu. Jego aktywacja przez leptynę („hormon sytości” wydzielany przez komórki tłuszczowe), a w mniejszym stopniu także przez insulinę, za pośrednictwem odpowiednich receptorów aktywuje wydzielanie pro-opiomelanokortyny. Dalsza przemiana w α- i β- melenokorytynę umożliwia aktywację receptora melanokortyny 4 (MS4R) w jądrze przykomorowym podwzgórza, co uruchamia sygnał sytości. Z kolei grelina (hormon wydzielany w przez komórki okładzinowe żołądka), pobudza łaknienie poprzez stymulację komórek wydzielających neuropeptyd Y (NPY) oraz białko z rodziny Agouti ( AGgRP) w jądrze łukowatym podwzgórza, które hamują działanie receptora MS4R i wyzwalają uczucie głodu. Aktywacja części sygnalizującej sytość jednocześnie hamuje działanie anoreksygenne melanokortyny.

Dotychczas opisano zaledwie kilkanaście rodzin z mutacjami genu leptyny LEP powodującymi utratę funkcji genu. Gen kodujący leptynę u człowieka, homologiczny do mysiego genu ob, zlokalizowany jest na długim ramieniu chromosomu 7q31.3 [11, 12, 13]. U osób z homozygotyczną mutacją tego genu obserwowano ciężką otyłość rozwijającą się wkrótce po urodzeniu, z towarzyszącą hiperfagią, zaburzeniami zachowania, zaburzeniem odporności komórkowej (niedobór limfocytów T) i ciężkimi infekcjami w wieku dziecięcym, hipogonadyzmem hipogonadotropowym, opóźnieniem dojrzewania i zaburzeniami płodności. W surowicy krwi stwierdzano u nich nieoznaczalne stężenie leptyny oraz hiperinsulinemię adekwatną do stopnia otyłości. Objawy towarzyszące otyłości wynikały z dodatkowych funkcji leptyny związanych z regulacją układu immunologicznego (hamowanie aktywności makrofagów i monocytów, regulacja fagocytozy i indukcja ekspresji cytokin, hamowanie proliferacji komórek T), oraz wpływu na obrót kostny oraz reprodukcję i termogenezę. Leptyna wpływa również na uwalnianie gonadoliberyny w podwzgórzu i reguluje wydzielanie hormonów przysadkowych – LH i FSH, przez co stymuluje pojawienie się cech pokwitania. Otyłość uwarunkowana mutacjami homozygotycznymi genu LEP dziedziczy się autosomalnie recesywnie, za czym przemawia fakt pokrewieństwa rodziców, w nielicznych przypadkach zidentyfikowanych rodzin z tego typu otyłością. Wykazano niezwykłą skuteczność podskórnego podawania rekombinowanej ludzkiej leptyny na normalizację apetytu (zmniejszenie pobierania pokarmu o 45-85%) oraz spadek masy ciała wywołany utratą tkanki tłuszczowej. Dramatyczną poprawę po 48 miesięcznym leczeniu leptyną uzyskano u 3 letniego chłopca ważącego 42 kg (>>97c), u którego nastąpił spadek masy ciała do 32 kg (75c) [12, 14, 15].

W komórkach podwzgórza znajduje się receptor leptyny, który pod wpływem leptyny inicjuje proces hamowania apetytu. W 1995 r. Tartaglia i wsp. [16] zidentyfikowali gen receptora leptyny u szczepu myszy (db/db) z otyłością spowodowaną opornością na leptynę w wyniku mutacji receptora tego genu. Receptor ten odgrywa kluczową rolę w transporcie leptyny przez barierę krew-mózg. Mimo zwiększonego stężenia krążącej leptyny, myszy wykazywały hiperfagię, obniżoną podstawową przemianą materii, insulinooporność hiperinsulinemię oraz cukrzycę typu 2. Podanie syntetycznej leptyny myszom db/ db, w przeciwieństwie do myszy ob/ob, nie skutkowało zmniejszeniem apetytu ani spadkiem masy ciała [12, 16]. U ludzi receptor leptyny kodowany jest przez gen Ob-R zlokalizowany na chromosomie 1p31.Większość mutacji genu receptora leptyny skraca część wewnątrzkomórkową lub transbłonową tego receptora [17]. U osób z homozygotyczną mutacją genu Ob-R, otyłość znacznego stopnia i nadmierny apetyt występują podobnie jak w przypadku niedoboru leptyny, ale towarzyszą im objawy niedoczynności przysadki z umiarkowanym zahamowaniem wzrastania, opóźnieniem dojrzewania i ośrodkową niedoczynnością tarczycy w wieku dziecięcym. W surowicy krwi stężenie leptyny jest zwykle prawidłowe, towarzyszy jej nieznaczna hiperinsulinemia adekwatna do otyłości, prawidłowy poziom kortyzolu, obniżony poziom GH i TSH oraz prawidłowa temperatura ciała. Patogenne mutacje LEPR wykryto u 3% osób z ciężką otyłością o wczesnym początku [18], ale powszechnie występujące warianty polimorficzne są obserwowane w skali całej populacji u osób z podwyższonym BMI [19].

Wyniki licznych badań nad patogenezą otyłości, szczególnie zapoczątkowanej w wieku dziecięcym doprowadziły do wykrycia wielu innych genów szlaku leptyna-melanokortyna w podwzgórzu. Wśród nich wymienia się takie geny jak: POMC, PSK1, MC4R, BDNF. Z ciężkimi postaciami dziecięcej otyłości koreluje się geny: NTRK2B oraz SIM1.

W 1998 roku H. Krude i wsp. opisali obecność homozygotycznych mutacji genu pro-opiomelanokortyny (POMC), stanowiącego prekursor dla wytwarzania hormonów, w tym adrenokortykotropiny (ACTH) i hormonu stymulującego melanocyty (MSH) u kilkorga dzieci z objawami wczesnej otyłości z nadmiernym apetytem oraz kryzą nadnerczową z hipoglikemią i hiponatremią w okresie noworodkowym. U osób pochodzenia europejskiego dodatkowo obserwowano objawy niedoboru melaniny pod postacią jasnej karnacji skóry i rudych włosów oraz niekiedy cholestazę i żółtaczkę [20]. W surowicy krwi osób chorych charakterystyczne jest niskie stężenie ACTH i kortyzolu, jednak substytucja hormonalna nie wpływa istotnie na obniżenie BMI [21]. Badania wielu grup autorów wykazały, że leptyna stymuluje ekspresję POMC w neuronach jądra łukowatego w podwzgórzu. POMC podlega potranslaacyjnej modyfikacji do wydzielania melanokortyny, która aktywuje receptory melanokortyny do modulowania różnych funkcji w centralnym układzie nerwowym, nadnerczach i skórze. Melanokortyny są agonistami (współdziałają) z receptorami. Brak proopiomelanokortyny spowodowany mutacjami genu *POMC*, skutkuje hiperfagią i wczesną otyłością w wyniku zaburzenia funkcji szlaku sygnałowego melanokortyny. Wśród najbliższych krewnych dzieci będących homozygotami mutacji *POMC* (z brakiem proopiomelanokortyny) zaobserwowano częstsze występowanie otyłości u heterozygot pod względem mutacji *POMC*. Sugeruje to, że nawet heterozygotyczne mutacje tego genu mogą również skutkować rozwojem otyłości o mniejszym nasileniu [12].

Mutacje genu receptora melanoktyny 4 (*MC4R*) zmapowanego w regionie 8q21 są najczęstszą przyczyną izolowanej otyłości z początkiem we wczesnym dzieciństwie i występują w 1-6% przypadków ekstremalnej otyłości u dzieci i młodocianych. W odróżnieniu od wcześniej opisanych, rozwój otyłości występuje u osób będących nosicielami heterozygotycznych mutacji tego genu (dziedziczenie autosomalne dominujące) [22]. U nosicieli mutacji genu receptora melanokortyny 4 (*MC4R*) dodatkowo stwierdza sie hyperinsulinemię oraz przyśpieszenie wzrastania. Nadmierny apetyt ujawnia się w pierwszym roku życia, natomiast z wiekiem staje się mniej nasilony [23]. Terapia dostępnymi lekami, agonistami MC4R jest obarczona istotnymi efektami niepożądanymi, wywołanymi aktywacją układu autonomicznego. Dlatego tego typu farmakoterapia nie znalazła zastosowania. Wśród rzadziej opisywanych genów związanych z otyłością, działających na poziomie podwzgórza, należy wymienić *PCSK1*, *SIM1*, *BDNF*, *NTRK2* oraz *GSHR*.

Mutacje genu *PCSK1* (proprotein convertase, subtilisin/kexin-type, 1, inna nazwa – gen prohormonu konwertazy1/3 (PC1/3) ) zmapowanego w regionie 5q15 zaburzają funkcję PC1/3 (prohormon konwertazy1), enzymu zaangażowanego w proces odszczepienia wielu prekursorów hormonów peptydowych, związanych z regulacją przyjmowania pokarmów, homeostazą glukozy i homeostazą energii, na przykład proopiomelanokortyną, proinsuliną, proglukagonem i proergreliną. Był on jednym z pierwszych genów korelowanych z monogeniczną otyłością wczesną. W sporadycznych przypadkach nosicieli homozygotycznych mutacji skutkujących utratą funkcji genu *PCSK1* obserwowano hiperfagię, wczesny rozwój otyłości, hipogonadyzm hipogonadotropowy, hipokortyzolemię, hipoglikemię poposiłkową, podwyższony poziom POMC, a we wczesnym wieku dziecięcym zaburzenia wchłaniania i przewlekłe ciężkie biegunki. Badania ostatnich lat dotyczących genomu w różnych populacjach wykazały ponadto silne sprzężenie pomiędzy polimorfizmami *PCSK1* i zwiększonym ryzykiem otyłości [24].

Uszkodzenie funkcji genu *SIM1 (*zmapowanego w regionie 6q16.3), wykazującego swoją aktywność transkrypcyjną w jądrze przykomorowym podwzgórza opisano u dziewczynki z powstałą *de novo* zrównoważoną translokacją między chromosomami 1p22.1 i 6q16.2. Hiperfagia, wczesny rozwój otyłości oraz podobieństwo fenotypowe do zespołu Pradera i Williego sugerowało rolę genu *SIM1* w patogenezie ciężkiej otyłości o wczesnym początku. [25]. Było to zgodne z wcześniejszymi doniesieniami w przypadkach delecji obejmujących region 6q16 skutkujących delecją genu *SIM1* i objawami skrajnej otyłości przypominającymi zespół Pradera i Williego. Badania funkcjonalne różnych zmian w genie *SIM1* potwierdzają istnienie związku przyczynowego utraty funkcji genu *SIM1* a objawami ciężkiej otyłością zarówno z obecnością jak i bez fenotypu podobnego do zespołu Pradera i Wiliego [26].

Mutacje genu *BDNF* (zmapowanego w regionie 11p14.1), zaangażowanego w synaptyczną funkcję przekazywania sygnału MC4R opisano u dziecka ze skrajną otyłością i nadmiernym apetytem, nadpobudliwością, upośledzonymi funkcjami poznawczymi oraz osłabioną pamięcią [27].

Mutacje genu *NTRK2*, genu kodującego receptor TrkB wykryto u dziecka z ciężką otyłością i nadmiernym apetytem oraz opóźnionym rozwojem, trudnościami w nauce, zaburzoną pamięcią krótkotrwałą oraz obniżonym progiem bólowym [28]. Opisano też mutację w genie receptora greliny *GSHR*, u osób z niskim wzrostem oraz stosunkowo późnym, w okresie dojrzewania, rozwojem otyłości [29].

Opisane powyżej przykłady genów o potwierdzonej roli w patogenezie otyłości o wczesnym początku nie wyczerpują zagadnienia związanego z przyczynami otyłości monogenowej – niesyndromicznej, a z uwagi na niezwykle rzadkie występowanie mutacji w tych genach nie mogą tłumaczyć problemu otyłości w skali populacji. Liczne badania populacyjne prowadzone w ciągu ostatniej dekady z zastosowaniem wysokoporzepustowego sekwencjonowania następnej generacji jak również badania funkcjonalne wykazują obecność wielu polimorficznych zmian w obrębie wymienionych powyżej genów jak również w nowych genach ściśle skorelowanych z ryzykiem otyłości zarówno o wczesnym początku jak i w wieku dojrzałym. Zestawienie skutków klinicznych omawianych genów otyłości monogenowej - niesyndromicznej przedstawiono w tabeli I.

**Tabela I:** Otyłość jednogenowa – przykłady korelacji genotypowo-fenotypowej. Table I. Monogenic obesity – examples of genotype-phenotype correlation.

Geny/Fenotyp	LEP	LEPR	POMC	MC4R	PCSK1	SIM1	BDNF	NTRK2	GRHB
Otyłość Hiperfagia	+	+	+	+	+	+	+	+	+
Niska Leptyna	+								
Infekcje	+	+							
Hipotyreoza		+							
Niedoczynność nadnerczy			+						
Hipoglikemia			+		+				
Zaburzenia jelitowe					+				
Wysoki wzrost				+					
rozwoju Zaburzenia psych.						+	+	+	
Niski wzrost		+							+

## Otyłość Zespołowa (Syndromiczna)

Otyłość zespołowa (syndromiczna) rozpatrywana jest w kontekście różnego rodzaju cech klinicznych powiązanych z określonymi cechami fenotypu, wadami wrodzonymi, niepełnosprawnością intelektualną oraz specyficznymi zaburzeniami zachowania. Z analiz Kaur i wsp. opublikowanych w 2017 roku przeprowadzonych na podstawie danych uzyskanych w różnych bazach, takich jak: MEDLINE, EMBASE, CINAHL, Pubmed, Orphanet, Web of Science oraz Cochrane Library databases wynika, że do 2016 roku, ukazało się aż 13.719 doniesień naukowych dotyczących otyłości zespołowej u ludzi. Na podstawie tego przeglądu wyłoniono 79 zespołów monogenowych z otyłością w obrazie klinicznym, z czego w 19 (24,1%) zespołów patologia molekularna warunkująca chorobę jest znana, w 11 (13,9%) częściowo poznana. Z pozostałej grupy 49 (62%) chorób, w 27 (34%) zmapowano potencjalne loci chromosomowe, zaś w pozostałych 22(27%) chorobach nie są dotychczas poznane, ani potencjalny gen, ani loci chromosomowe warunkujące wystąpienia określonych objawów. Trudności w ustaleniu etiologii tych zespołów w dużej mierze wynikają z niezwykle rzadkiego ich występowania w populacji oraz w wielu przypadkach złożonego mechanizmu dziedziczenia, podlegającego regulacji poprzez czynniki epigenetyczne, mozaikowość czy też współdziałanie genów modyfikujących. Oszacowanie częstości występowania było możliwe tylko dla 12 spośród 79 zespołów i wynosi ona od 1:565 do <1:1,000,000 wśród żywo urodzonych. Rozwój nowoczesnych technik diagnostycznych, technik molekularnych, a także technik obrazowych (np. funkcjonalny rezonans magnetyczny fMRI czy PET) przyczynia się do lepszego poznania, które części mózgu i potencjalne geny wykazujące w nim swoją ekspresję odpowiadają za zaburzenie homeostazy energetycznej, hiperfagię, wzrost BMI i rozwój otyłości. W przyszłości może to przyczynić się do ustalenia korelacji fenotypowo - genotypowej różnych postaci otyłości i opracowania celowanych leków do jej skutecznego leczenia [30].

Otyłość zespołowa uwarunkowana jest obecnością mutacji określonego genu (ów) dziedziczonymi autosomalnie dominująco lub sprzężonych z chromosomem X, aberracjami chromosomowymi, zmianami liczby kopii fragmentów DNA (Copy Number Variations − CNVs) w zespołach mikrodelecji/mikroduplikacji określanych mianem tzw. chorób genomowych. Co ciekawe, w niektórych spośród tych zespołów obserwuje się dużą heterogenność genetyczną czyli występowanie bardzo podobnego fenotypu klinicznego mimo zróżnicowanego, wielogenowego uwarunkowania. Plejotropowy charakter skutków klinicznych określonej zmiany w genomie powoduje, że diagnostyka tych zespołów na poziomie klinicznym nierzadko jest bardzo trudna i wymaga ścisłej współpracy wielu specjalistów, w szczególności w zakresie biologii molekularnej.

Do najlepiej poznanych zespołów otyłości syndromicznej należą: zespół Pradera i Williego, zespół Bardeta i Biedla, zespół Cohena, zespół Börjeson i Lehmana, zespół Alströma, zespół Simpsona i Golabiego, zespół Carpentera, zespół Wilsona i Turnera, zespół Smitha i Magenis, zespół disomii chromosomu 14 (z. Temple), osteodystrofia Albrighta typu 1 oraz niektóre zespoły mikrodelecji i mikroduplikacji przedstawione w tabeli II.

**Tabela II:** Wybrane zespoły genetyczne z otyłością w obrazie klinicznym. Table II. Examples of syndromes with obesity in clinical picture.

Nazwa zespołu/genu i OMIM	Częstość występowania	Objawy kliniczne	Czy otyłość jest zawsze objawem?	Czy znane jest podłoże genetyczne?	Typ dziedziczenia	Gen/chromosom
Zespół Albrighta pseudo-hipoparatyroidizm typ la (OMIM#103580)	nieznana	Skrócenie środkowych paliczków palców dłoni (brachymetaphalangism), niskorosłość, otyłość, niepełnosprawność intelektualna	Tak	Tak	Autosomalne dominujące	*GNAS1/GNAS*
Zespół Alströma (OMIM#203800)	<l/milion Katagiri et al.	Wrodzona dystrofia siatkówki, utrata wzroku, niedosłuch, otyłość, insulinooporność, cukrzyca typu 2	Nie	Tak	Autosomalne recesywne	*ALMS1*
Zespół Bardeta-Biedla (OMIM#209900)	1/13,500-175,000 White et al.	Zwyrodnienie barwnikowe siatkówki, dysfunkcja nerek, otyłość, polidaktylia, zaburzenia zachowania, hipogonadyzm	Nie	Nie	Autosomalne recesywne	*BBS1 - BBS21/C8ORF37* -21 zidentyfikowanych genów
Zespół Börjesona -forsowana-Lehmanna (OMIM#301900)	nieznana	niepełnosprawność intelektualna znacznego stopnia, padaczka, małogłowie, nieskorosłość, otyłość, hipogonadyzm, ginekomastia	Nie	Tak	Sprzężone z chromosomem X	*PHF6*
Zespół Carpentera/ Akrocefalopolisyndactylia t.ll (OMIM#201000)	1/milion Victorine et al.	Akrocefalia, syndaktylia skórna palców, brachydaktylia lub agenezja paliczków środkowych palców dłoni i stóp, preaksjalna polidaktylia, wrodzona wada serca, niepełnosprawność intelektualna, hypogenitalizm, otyłość, przepuklina pępkowa	Nie	Tak	Autosomalne recesywne	*RAB23*
Zespół Cohena (OMIM#216550)	1/565 to 2000 Murphy et al.	niepełnosprawność intelektualna cechy dysmrofii, małogłowie, dystrofia siatkówki, otyłość tułowiowa, wiotkość stawów, okresowa neutropenia	Nie	Tak	Autosomalne recesywne	*VPS13B/COH1*
Zespół Pradera-Willego (OMIM#176270)	1/20,000 Reinhardt et al.	Zaburzenie rozwoju somatycznego i trudności w karmieniu w wieku niemowlęcym, otyłość i hiperfagia od wczesnego dzieciństwa, obniżone napięcie mięśniowe, hipogonadyzm, niedobór hormonu wzrostu, niski wzrost, małe dłonie 1 stopy, zaburzenia zachowania, niepełnosprawność intelektualna	Tak	Nie	Różne defekty genetyczne	Zaburzenia imprintingu MKRN3/ZNF127, MAGEL2, Delecja NDN, Utrata funkcji NPAP1/C15orf2, Utrata funkcji ojcowskiej kopii SNURF-SNRPN
Zespoły podobne do Pradera-Willego (Prader-Willie-like phenotype)	nieznana	Opóźnienie rozwoju psychoruchowego, otyłość, hipotonia, skrócenia kończyn	Tak	Nie	Autosomalne dominujące Sprzężone z chromosomem X dominujące	*SIM1, MRAP2, 6ql6.3q23.3* duplications *FMR1*
Zespól Smith-Magenis (OMIM#182290)	1/15,000-25,000 Carmon Mora et al.	Niepełnosprawność intelektualna, opoźnienie rozowju mowy, dysmorfia, zaburzenia snu, otyłość, zaburzenia zachowania	Tak	Tak	Autosomalne dominujące	*RAI1*
Zespól MEHMO (OMIM#300148)	nieznana	Niepełnosprawność intelektualna padaczka, hipogonadyzm, otyłość, małogłowie	Tak	Nie	Mitochondrialne sprzeżone z chromosomem X	mikrodelecje
Zespół MIMO (OMIM#157980)	nieznana	Makrocefalia, otyłość, wady gaiki ocznej (szczelina siatkówki, oczopląs), opóźniony wiek kostny, niepełnosprawność intelektualna	Tak	Tak	Autosomalne dominujące	nieznane
Zespól Downa	1/650-1000	Opóźnienie rozwoju psychoruchowego, niepełnosprawność intelektualna, wada serca, niskorosłość, obniżone napięcie mięśniowe, zespól cech dysmorfii, niedoczynność tarczycy, otyłość, białaczka	Nie	Tak	Autosomalne dominujące	Trisomia 21, Mozaikowa trisomia 21, translokacje chromosomu 21
Zespół Tempie (OMIM#616222)	nieznana	Niska masa ciała urodzeniowa/IUGR, niskorosłość, otyłość tułowiowa, wiotkość stawowa, małe dłonie, opoźnienie rozwoju ruchoweo i intelektualnego, rozwoju mowy przedwczesne dojrzewanie	Tak	Tak	Różne defekty genetyczne	UPD 14 mat, Mutacja epigenetyczna, delecja ojcowska
Zespół monosomii 1p36	~1/5000	Obniżone napięcie mięśniowe, opóźnienie rozwoju psychoruchowego i somatycznego, otyłość, zespół cech dysmorfii, małe dłonie i stopy	Tak	Tak	Autosomalne dominujące	mikrodelecje 1p36
Zespół proksymalnej delecji 16p11.2	nieznana	Otyłość, autyzm, opóźnienie rozwoju psychoruchowego, niepełnosprawność intelektualna, wady wrodzone	Tak	Nie	Autosomalne dominujące	*SH2B1, KCTD13*
Zespół dystalnej delecji 16p11.2	nieznana	Opóźnienie rozwoju psychoruchowego, zaburzenia zachowania, zespół cech dysmrofii, otyłość	Nie	Nie	Autosomalne dominujące	Mikrodelecje 16p11.2
Zespół mikroduplikacji 5p13	nieznana	Opóźnienie rozwoju psychoruchowego, zachowania autystyczne, otyłość, obrzęki limfatyczne, nadciśnienie tętnicze, makrocefalia	Nie	Tak	Autosomalne dominujące	*NIPBL*

**Zespół Pradera i Williego (PWS) (OMIM** − **1762700)** jest najczęstszą przyczyną genetycznie uwarunkowanej otyłości oraz modelowym przykładem choroby uwarunkowanej rodzicielskim piętnowaniem genomowym („genomic imprinting”). W ostatnich latach stał się również chorobą modelową do badania mechanizmów regulacji łaknienia i rozwoju otyłości u człowieka.

Wśród głównych objawów PWS występuje: znacznego stopnia hipotonia mięśniowa w okresie noworodkowym i niemowlęcym, brak/słaby odruch ssania, trudności w karmieniu, brak/słaby przyrost masy ciała w okresie niemowlęcym, hiperfagia, otyłość, niedobór wysokości ciała, małe dłonie i stopy, gęsta lepka ślina zasychająca w kącikach ust, cechy dysmorfii twarzy, hipogonadyzm hipogonadotropowy, opóźnienie rozwoju psychoruchowego oraz specyficzny typ zaburzeń zachowania związanych z hiperfagią (upór, wybuchy złości, natręctwa, skubanie skóry). Do niedawna opisywano 2 fazy zaburzeń odżywiania w PWS: okres niemowlęcy z trudnościami w karmieniu i okres hiperfagii z rozwojem otyłości powyżej 3 roku życia [31].

Z ostatnich badań wynika, że przejście z fazy słabego łaknienia i niedoboru masy ciała do fazy hiperfagii jest procesem złożonym i składa się z 7 faz z początkiem już w okresie płodowym. Zwykle do drugiego roku życia rozwój fizyczny jest odpowiedni do wieku, natomiast pomiędzy 2,1–4,5 rokiem życia następuje nadmierny przyrost masy ciała bez wzrostu łaknienia ani zwiększonej podaży kalorii. Wzmożone łaknienie (hiperfagia) i nadmierny przyrost masy ciała rozwija się zwykle pomiędzy 4-8 rokiem życia, natomiast faza niepohamowanego łaknienia następuje powyżej 8 lat. Fakty te powinny być uwzględnione w leczeniu dietetycznym i profilaktyce otyłości począwszy od okresu niemowlęcego [32]. Hiperfagia w PWS ma związek z nieprawidłową percepcją uczucia sytości. W porównaniu do osób zdrowych chorzy kończą jedzenie później, ale wkrótce (około ½ godziny po zakończeniu posiłku znowu pojawia się uczucie głodu). Rozwojowi otyłości w PWS sprzyja szereg czynników takich jak: zmniejszone zapotrzebowanie kaloryczne (60% w porównaniu do osób zdrowych), obniżona percepcja bólu żołądka po przejedzeniu, brak skłonności do wymiotów/ brak dyskomfortu po przejedzeniu, brak aktywności ruchowej, brak kontroli diety, zaburzenia zachowania związane z jedzeniem. Bez stałego nadzoru osób trzecich, chory nie jest w stanie utrzymać reżimu dietetycznego, co prowadzi do nadwagi 200-300%.

Badania nad patogenezą otyłości w PWS prowadzone od wielu lat lecz jak dotąd nie doprowadziły do ostatecznego wyjaśnienia przyczyny hyperfagii. Wiadomo, że jest to proces bardzo złożony, związany z zaburzeniem ośrodkowej regulacji osi podwzgórze – przysadka, w tym funkcji neuroprzekaźników związanych z regulacją ośrodków głodu i sytości w podwzgórzu.

Potwierdzają to wyniki badań z zastosowaniem funkcjonalnego MRI i PET w trakcie oglądania wysokokalorycznego pożywienia oraz po posiłku, które wykazały brak aktywacji regionów odpowiedzialnych za uczucie sytości po spożyciu posiłku oraz zwiększoną aktywację regionów odpowiedzialnych za uczucie głodu i motywacji (hipokamp oraz kora oczodołowo–czołowa) [33].

Stężenie greliny u chorych z PWS, tzw. „hormonu głodu” wytwarzanego przez komórki okładzinowe żołądka jest stale na bardzo wysokim poziomie, 3- krotnie wyższym niż u zdrowych osób. Obniżenie stężenia greliny po posiłku nie skutkuje jednak zmniejszeniem uczucia głodu. W jądrach przykomorowych podwzgórza u chorych z PWS stwierdza się zmniejszoną liczbę i objętość neuronów produkujących oksytocynę, która jest neuropeptydem hamującym łaknienie. Wyniki badań doświadczalnych zarówno na modelach mysich jak i u chorych z PWS sugerują, że mała dawka donosowo podawanej oksytocyny jest bezpieczna i może wpływać na redukcję apetytu oraz poprawę w zakresie zależnych od hiperfagii zaburzeń zachowania [34].

W patogenezie PWS odgrywają rolę 3 główne defekty molekularne: delecje w regionie 15q11.2-13 (~75% przypadków), matczyna disomia 15 (mUPD15) w około ~24% przypadków oraz defekty imprintingowe (~1-3% przypadków). Mimo wielu lat badań nadal nie udało się ustalić genów odpowiedzialnych za ekspresję kluczowych dla zespołu objawów. W ostatnich latach sugeruje się znaczenie delecji (podlegającego imprintingowi) skupiska genów SNORD-116, kodujących małe jąderkowe RNA (snoRNA – small nucleolar RNA) jako kluczowego genu odpowiedzialnego za hiperfagię i otyłość w PWS [35].

U chorych z zespołem Pradera i Williego od 2006 roku w Polsce z powodzeniem stosuje się terapię rekombinowanym hormonem wzrostu (rGH) (od 2 roku życia), co pozwala na poprawę bilansu energetycznego, obniżenie całkowitej masy ciała przy jednoczesnym zwiększeniu beztłuszczowej masy ciała (zwiększenie siły mięśniowej), a w konsekwencji przyspieszenie rozwoju psychoruchowego uzyskanie wzrostu zbliżonego do rówieśników, spektakularną normalizację fenotypu oraz poprawę jakości życia. [31, 36, 37, 38].

**Zespół Bardeta i Biedla (BBS) (OMIM – 209900)** jest rzadkim zespołem, dziedziczonym autosomalnie recesywnie, w którym otyłości centralnej rozwijającej się na przełomie 1 i 2 roku życia, towarzyszą: niepełnosprawność intelektualna i zmienne problemy z zachowaniem, polidaktylia pozaosiowa dłoni i stóp (68-80% chorych) oraz hipogonadyzm i hipogenitalizm u pacjentów płci męskiej. Średnie BMI szacuje się na 31,5–36,6 kg/m^2^. W późniejszym okresie rozwija się postępująca dystrofia siatkówki lub zwyrodnienie barwnikowe (90% chorych), prowadzące często do ślepoty nocnej u młodych dorosłych. W obrazie choroby stwierdza się wady układu kielichowo-miedniczkowego oraz torbielowatość nerek, oraz zwykle łagodną ich niewydolnością, a także wrodzone wady serca, kardiomiopatię, nadciśnienie tętnicze oraz cukrzycę z insulinoopornością i nietolerancją glukozy.

Zespół Bardeta i Biedla należy do tzw. grupy ciliopatii i charakteryzuje się heterogennością genetyczną – jest uwarunkowany zmianami w co najmniej 20 różnych genach: BBS1, BBS2, ARL6 (BBS3), BBS4, BBS5, MKKS (BBS6), BBS7, TTC8 (BBS8), BBS9, BBS10, TRIM 32 (BBS11), BBS12, MKS1 (BBS13), CEP290 (BBS14), WDPCP (BBS15), SDCCAG8 (BBS16), LZTFL1 (BBS17), BBIP1 (BBS18). Najczęściej mutacje stwierdza się w genach: BBS1 – 23,1%, BBS10 – 20%, BBS2 – 8,1%, BBS9 – 6% oraz MKKS (BBS6) – 5,8%. W około 20% przypadków nie identyfikuje się mutacji w żadnym z wymienionych genów [39]. Zazwyczaj BBS dziedziczy się jako cecha autosomalna recesywna, ale w kilkunastu rodzinach (19 z typem 2 BBS oraz 9 z typem 6 BBS) stwierdzano tzw. dziedziczenie trójalleliczne. Sugerowano na tej podstawie, że BBS jest chorobą o kompleksowym wielogenowym uwarunkowaniu, a do pełnej ekspresji cech klinicznych konieczna jest obecność dodatkowej trzeciej mutacji w innym locus.

**Zespół Alströma (OMIM – 203800)** jest rzadkim zespołem zaliczanym do grupy ciliopatii uwarunkowanym mutacjami w genie ALMS1 zmapowanym w regionie 2p13.1. Dziedziczy się autosomalnie recesywnie. Chorych charakteryzuje hiperfagia pojawiająca się już w wieku niemowlęcym, której towarzyszy hiperinsulinomia o nasileniu nieproporcjonalnym w stosunku do otyłości, przewlekła hiperglikemia, a następnie cukrzyca typu 2 oraz umiarkowana otyłość brzuszna. Objawami przepowiadającymi wystąpienie prowadzącej od ślepoty neuropatii z degeneracją czopków i pręcików mogą być oczopląs i światłowstręt obecne już w pierwszym roku życia. Powoli postępujący niedosłuch zmysłowo-nerwowy wykrywany jest z reguły około 5 roku życia. U części chorych już w pierwszych latach życia jest diagnozowana kardiomiopatia, zaś w drugiej lub trzeciej dekadzie postępująca nefropatia [40].

**Zespół Cohena (OMIM – 216550)** jest dziedziczony autosomalnie recesywnie i uwarunkowany mutacjami w genie VPS13B zmapowanym w regionie 8q22.2. Stosunkowo częste występowanie choroby stwierdza się w populacji Żydów Ashkenazyjskich oraz Finlandii. Osoby dotknięte tym zespołem zwykle cechuje opóźnienie rozwoju psychoruchowego z hipotonią i nadmierną wiotkością stawów w okresie niemowlęcym i wczesnodziecięcym, niepełnosprawność intelektualna w stopniu umiarkowanym bądź znacznym, często brak/znaczne opóźnienie rozwoju mowy, padaczka (występująca prawie u wszystkich chorych), małogłowie, ataksja, charaktery-styczny fenotyp behawioralny (miła i pogodna osobowość), niski wzrost, smukłe, długie palce. dłoni, wąskie stopy. Wśród charakterystycznych cech dysmorfii, wymienia się takie jak: twarz hipotoniczna, wysoki grzbiet nosa, brwi o falistym kształcie, cienka warga górna, tendencja do otwartych ust, wysunięcie do przodu górnych siekaczy. Po piątym roku życia stwierdza się postępującą krótkowzroczność często skojarzoną z dystrofią siatkówki i naczyniówki. W badaniach obrazowych stwierdza się hipoplazje móżdżku oraz duże ciało modzelowate. Ważnym diagnostycznie objawem jest okresowa leukopenia/neutropenia o łagodnym nasileniu, często już w okresie niemowlęcym. Otyłość brzuszna średniego stopnia, pojawia się koło 5 roku życia [41].

**Zespół Börjesona, Forssmana i Lehmanna (OMIM – 301900)** jest bardzo rzadkim zespołem dziedziczącym się recesywnie w sposób sprzężonym z chromosomem X. Przyczyną choroby są mutacje w genie PHF 6 zmapowanym w regionie Xq26.2. Niemowlęta płci męskiej mogą wykazywać objawy fenotypowe przypominające zespół Pradera i Williego (hipotonia, trudności w karmieniu oraz hipogenitalizm). Wśród cech dysmorfii wymienia się: pogrubienie rysów twarzy, duże odstające małżowiny uszne, głęboko osadzone gałki oczne, wąskie szpary powiekowe, szerokie rozstawienie palców stóp. Zwracają uwagę mięsiste dłonie, hipoplazja dystalnych i środkowych paliczków dłoni (palce są charakterystycznie zwężone na końcach i wiotkie w stawach międzypaliczkowych). Niepełnosprawność intelektualna zwykle umiarkowanego lub znacznego stopnia, małogłowie, padaczka, niskorosłość, skolioza, kifoza, krótkowzroczność oraz u części pacjentów cechy polineuropatii czuciowo-nerwowej dopełniają obraz kliniczny choroby. Znacznego stopnia otyłość z towarzyszącą nasiloną ginekomastią rozwija się dopiero w późnym dzieciństwie, a w wieku dojrzałym jest zwykle znacznego stopnia. Matki chorych mężczyzn będące nosicielkami mutacji genu PHF6 mogą wykazywać dyskretne cechy dysmorfii z pogrubieniem rysów twarzy [42].

**Zespół Albrighta (OMIM – 103580)**, inne nazwy zespołu: Albright hereditary osteodystrophy (AHO), rzekoma niedoczynność przytarczyc typu 1a, dziedziczna osteodystrofia Albrighta, jest grupą chorób metabolicznych, charakteryzujących się opornością tkanek docelowych na parathormon. Wyróżnia się cztery typy choroby: Ia, Ib, Ic i II. Najbardziej znana jest postać 1A, choroba dziedziczona w sposób autosomalny dominujący spowodowana mutacjami w genie GNAS1, zmapowanym w regionie 20q13.32, który koduje podjednostkę alfa białka Gs. Ekspresja objawów związana jest z rodzicielskim piętnowaniem genomowym (matczyny imprinting genomowy). Chorych cechuje zauważalna około 3-5 roku życia niskorosłość, charakterystyczna, okrągła twarz, pełne policzki, opóźnione wyrzynanie zębów, hipoplazja szkliwa i wady zgryzu, wady kostne z krótką szyją, krótkimi kośćmi śródręcza i śródstopia oraz palcami (zwłaszcza 4 i 5), osteopenia i deformacje kości długich, niekiedy kraniosynostoza i zgrubienie pokrywy czaszki, ektopiczne kostnienie tkanek miękkich oraz skrócanie dystalnego paliczka kciuka (od trzeciej do piątej kości śródręcza), napady tężyczki i parestezji, połączone z opornością na działanie hormonu wzrostu, tyreotropiny, gonadotropin. W części przypadków występuje niepełnosprawność intelektualna [43].

**Zespół Carpentera (acrocephalopolysyndactyly type II) (OMIM – 201000)** uwarunkowany jest mutacjami w genie RAB23 (zmapowanyw regionie 6p12.1) lub genie MEGF8 (zmapowany w regionie 19q13.2), dziedziczy się autosomalnie recesywnie. W obrazie choroby występują takie cechy jak: makrocefalia (wieżowaty kształt czaszki spowodowany nieprawidłowym zarastaniem szwów czaszkowych), syndaktylia skórna palców dłoni i stóp, polidaktylia przedosiowa (dodatkowe palce od strony kciuka), niedosłuch przewodzeniowy, wrodzone wady serca i niepełnosprawność intelektualna. Otyłość występuje u większości chorych już w pierwszych latach życia [44].

**Zespół łamliwego chromosomu X, FraX (OMIM – 300624)** jest spowodowany nadmierną (>200) ekspansją niestabilnych powtórzeń trójnukleotydowych CGG w promotorze genu FMR1, zmapowanym w regionie Xq27.3. Należy do najczęstszych po zespole Downa przyczyn niepełnosprawności intelektualnej u płci męskiej. Choroba dziedziczy się dominująco w sposób sprzężony z chromosomem X. Charakterystyczną pełną ekspresję objawów zespołu FraX obserwuje się u osób płci męskiej (niepełnosprawność intelektualna, cechy nadpobudliwości psychoruchowej, spektrum zachowań autystycznych, specyficzna dysmorfia twarzy w wieku dojrzałym, makroorchidyzm po okresie dojrzewania). Kobiety nosicielki mutacji genu FMR1 wykazują łagodną ekspresję objawów pod postacią trudności szkolnych (w około 50% przypadków). U niektórych chorych występuje ponadto nadmierny apetyt, otyłość i niskorosłość. Co ciekawe, otyłość występuje także u osób z delecjami obejmującymi gen FMR1 [45].

**Zespół MEHMO (Mental retardation – niepelnosprawność intelektualna), Epileptic seizures – padaczka, Hypogenitalism – hipogenitalizm, Microcephaly – małogłowie, Obesity – otyłość) (OMIM – 300148)** należy do bardzo rzadko występujących zespołów otyłości sprzężonej z chromosomem X, a jego nazwa jest akronimem wiodących objawów zespołu. Locus genu choroby znajduje się najprawdopodobniej w regionie Xp21.1-p22.13, natomiast gen warunkujący objawy nie jest dotychczas poznany. Znacznego stopnia otyłość rozwija się w niemowlęctwie. Niekiedy opisywane są znaczne opóźnienie rozwoju, niski wzrost, wzmożone napięcie mięśniowe, wygórowane odruchy ścięgniste, oczopląs, nadmierna pobudliwość. Zwykle chorzy chłopcy umierają po 2 roku życia. Badania laboratoryjne wskazują na zaburzone działanie kompleksów 1, 3 i 4 łańcucha oddechowego w mitochondriach. Nieprawidłowe, powiększone mitochondria widoczne w obrazach z mikroskopii elektronowej kwalifikują zespół jako chorobę mitochondrialną [46].

## Otyłość w Aberracjach Chromosomowych

Badanie chromosomów człowieka umożliwiło poznanie przyczyn znanych wcześniej chorób genetycznych. Stopniowy rozwój technik badania chromosomów, w tym FISH (@uorescencyjna hybrydyzacja in situ) i mikromacierzy (aCGH), umożliwił wykrywanie submikroskopowych rearanżacji chromosomowych, takich jak mikrodelecje i mikroduplikacje chromosomowe. Skutkiem mikrodelecji/mikroduplikacji chromosomowych jest brak funkcji/nadekspresja wielu przyległych genów znajdujących się w regionie utraconego lub zduplikowanego odcinka chromosomu. Skutki kliniczne zależą od wielkości zmiany, i jej zawartości genetycznej, a w niektórych mikrodelecjach od ich rodzicielskiego pochodzenia (choroby imprintingowe) − np. zespół delecji 15q11-13. W większości przypadków zmiany tego typu powstają de novo, natomiast w niektórych są one produktem zrównoważonych rearanżacji chromosomowych, odziedziczonych od jednego z rodziców.

**Trisomia chromosomu 21 pary w zespole Downa** – typowym cechom zespołu (niepełnosprawność intelektualna, obniżone napięcie mięśni, zaburzenia hormonalne, dysmorfia i wady wrodzone) towarzyszy otyłość, w okresie dziecięcym u około 15-50%, a w wieku dorosłym u 45-90% chorych. Czynnikiem sprzyjającym otyłości jest zmniejszona aktywność fizyczna wynikająca z hipotonii mięśniowej, jak i zmniejszone spoczynkowe zużycie energii. U dzieci z zespołem Downa obserwowano podwyższone stężenie leptyny, wydaje się też, że do otyłości może istotnie przyczyniać się niedoczynność tarczycy [47].

**Zespół mikrodelecji 1p36 (OMIM** − **607862)** – w obrazie klinicznym stwierdza się cechy niepełnosprawności intelektualnej zwykle w stopniu głębokim, brak ekspresji mowy, zaburzenia zachowania (agresja), mnogie wady strukturalne, głównie mózgu (małogłowie u 38% chorych) i serca (kardiomiopatia rozstrzeniowa, tetralogia Fallota, zespół Ebsteina u 45-70% chorych), duże późno zarastające ciemię przednie cechy dysmorfii oraz otyłość w wieku dojrzałym [48].

**Zespół mikrodelecji 16p11.2 (OMIM** − **611913)** – jedna z pierwszych wykrytych mikrodelecji identy-fikowana jest u około 0,3-0,7% pacjentów z cechami niepełnosprawności intelektualnej i zaburzeń zachowania. W jej obrazie klinicznym dominują: opóźnienie rozwoju intelektualnego, zaburzenia poznawcze i behawioralne oraz zaburzenia zachowania (nadpobudliwość psychoruchowa ze skłonnością do agresji, zaburzeń kompulsywnych), zaburzenia ze spektrum autyzmu, choroby psychiatryczne (schizofrenia, choroba dwubiegunowa). W przypadku objęcia delecją genu SH2B1, zaangażowanego w szlak sygnałowy leptyny, dodatkowo pojawia się nadmierny apetyt, wcześnie rozwijająca się otyłość i ciężka insulinooporność [49].

**Zespół mikrodelecji 6q16.2**, obejmuje gen SIM1. Obraz kliniczny przypomina zespół Pradera i Williego (Prader-Willi-like phenotype), z hipotonią i trudnościami w karmieniu w okresie noworodkowym z następową otyłością i głębokiego stopnia niepełnosprawnością intelektualną oraz krótkimi kończynami, którym niestale towarzyszą: nadmierny apetyt, wady serca, nieznaczna dysmorfia twarzowa [50].

Inne nowo opisane zespoły mikrodelecyjne są związane z regionem 13q34, z opóźnionym wzrastaniem i rozwojem psychoruchowym, łagodną dysmorfią twarzową, otyłością znacznego stopnia i niepełnosprawnością intelektualną [51] oraz z regionem 12q24.21, w którego przypadku opisano hipotonię, cechy dysmorfii, znaczące zwiększenie masy ciała we wczesnym wieku dziecięcym, znaczne opóźnienie rozwoju i wolne tempo rozwoju, a niekiedy także wolny rozwój mowy i zaburzenia zachowania [52] lub cechy zespołu Holta i Orama z towarzyszącym niskim wzrostem, otyłością i opóźnionym dojrzewaniem [53].

**Zespół mikroduplikacji 7q11.23 (OMIM** − **609757)**, obejmującej region krytyczny dla zespołu Williamsa. Nadekspresja genu YWHAG zlokalizowanego w regionie duplikacji jest związana z otyłością, opóźnieniem rozwoju mowy, łagodną niepełnosprawnością intelektualną, niekiedy wadami serca lub OUN, a często problemami behawioralnymi, w tym ADHD, niepokojem związanym z kontaktami społecznymi, zaburzeniami ze spektrum autyzmu [54]. W 2016 roku opisano 5 przypadków mikroduplikacji w regionie 17p13.1, z cechami niepełnosprawności intelektualnej, zróżnicowanymi cechami dysmorfii (zwykle pełne policzki), niskim wzrostem, otyłością oraz różnorodnymi zaburzeniami hormonalnymi, w tym najczęściej cukrzycą [55].

Otyłość jest też opisywana jako cecha w innych zespołach mikrodelecyjnych, w tym 2p25, 2q37, 3pter, 7q22.1, 9q34, 10p15.3, 14q11.2, 14q12, 10q22, Xq27, a także z zespołach mikroduplikacji 6q21-22, 6q27, 14q11.2 i Xq21 [56]. W tabeli II przedstawiono częściej występujące zespoły otyłości syndromicznej uwarunkowane monogenowo oraz obecnością defektów na poziomie chromosomowym.

Na rycinie 1 przedstawiono schemat postępowania diagnostycznego w przypadku podejrzenia otyłości syndromicznej.

**Ryc. 1:**
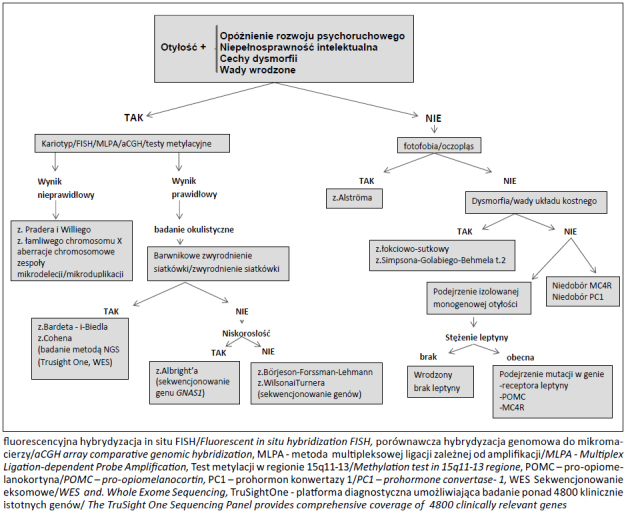
Algorytm diagnostyczny w przypadku podejrzenia zespołów z otyłością wg modyfikacji Farooqi S. (Best Practice & Research Clinical Endocrinology & Metabolism Vol. 19, No. 3, 359-374). Fig. 1. Diagnostic algorithm in the case suspected of monogenic syndrome with obesity. Modification according to Farooqi S. (Best Practice & Research Clinical Endocrinology & Metabolism Vol. 19, No. 3, 359-374)

Schemat postępowania diagnostycznego w przypadku podejrzenia otyłości syndromicznej powinien uwzględniać szczegółową analizę rodowodu i ocenę kliniczną (z określeniem wieku pojawienia się otyłości, wyników pomiarów antropometrycznych, oceny cech dysmorfii) oraz analizę wyników badań dodatkowych: (hormonalnych, biochemicznych, obrazowych), ocenę okulistyczną celem ukierunkowania procesu diagnostycznego. Wśród badań genetycznych w diagnostyce otyłości należy uwzględnić kariotyp, FISH, testy metylacyjne, aCGH oraz sekwencjonowanie określonych genów odpowiedzialnych za ekspresję objawów podejrzewanego zespołu monogenowego. W przypadku, kiedy na podstawie oceny cech klinicznych, rodowodowych, wyników badań biochemicznych, hormonalnych i radiologicznych istnieje podejrzenie wystąpienia choroby o wielogenowym uwarunkowaniu (np. zespół Bardeta i Biedla) można rozważyć wykonanie badań metodą sekwencjonowania następnej generacji (NGS) z zastosowaniem WES/zestaw TruSight One. W niektórych przypadkach należy zastosować kilka uzupełniających się metod diagnostycznych (np. w przypadku podejrzenia zespołu Pradera i Williego, zespołów mikrodelecji/mikroduplikacji) w celu ustalenia typu defektu molekularnego odpowiedzialnego za wystąpienie cech klinicznych choroby. Wyniki przeprowadzonych badań staną się podstawą do weryfikacji molekularnej rozpoznania klinicznego oraz oszacowania ryzyka genetycznego w rodzinie, co jest nieodłącznym elementem poradnictwa genetycznego.

## Otyłość Powszechna (Wieloczynnikowa, Wielogenowa)

W dalszych badania nad genetycznym uwarunkowaniem otyłości stosowano trzy różne metody poszukiwań. Pierwszą było typowanie genów kandydackich związanych z regulacją metabolizmu w oparciu o badania na zwierzętach lub in vitro. Weryfikacja wytypowanych genów polega na porównaniu częstości występowania mutacji danego genu w grupie osób z daną cechą (w tym przypadku otyłością) a występowania mutacji danego genu w grupie osób zdrowych. Ten sposób był z powodzeniem stosowany w identyfikacji genów odpowiadających za powszechne choroby i cechy złożone. Jednak w przypadku otyłości nie uzyskano zadawalających rezultatów, mimo przebadania ponad 200 genów związanych z regulacją apetytu, metabolizmem glukozy i tłuszczów oraz rozwojem tkanki tłuszczowej [57].

Drugą metodą była analiza sprzężeń polimorfizmów mikrosatelitarnych rozsianych regularnie w całym genomie z regionami wiążącymi się ze zwiększonym ryzykiem choroby. Ten sposób umożliwił identyfikację licznych genów odpowiedzialnych za choroby jednogenowe, jednak zestawienie wyników z 37 badań na ponad 31 tysiącach osób nie wskazało na istnienie głównego genu odpowiedzialnego za otyłość u ludzi [58]. Na ogół w badania wykazywały jedynie umiarkowany związek zidentyfikowanych wariantów z otyłością, a w większości przypadków inne badania nie potwierdzały wcześniej uzyskanych wyników.

Bardziej obiecujące wyniki przyniosło zastosowanie asocjacji polimorfizmów pojedynczych nukleotydów (GWAS, Genome-Wide Association Studies). W tej metodzie wykorzystuje się dziesiątki tysięcy polimorficznych wariantów DNA ograniczonych do pojedynczych nukleotydów (SNP) w celu wykrycia różnic w częstości występowania u osób chorych i zdrowych. Kolejne etapy pozwoliły wyróżnić kilkadziesiąt genów lub regionów wyraźnie związanych z otyłością. Pierwszy z nich ujawnił związek wariantu genu FTO (Fat mass- and Obesity-associated), w drugim etapie wykryto związek SNP w pobliżu genu MC4R. W kolejnej turze badań powiązano otyłość z polimorfizmem nukleotydów w obrębie lub w pobliżu genów: SH2B1, KCTD15, TMEM18 oraz NEGR1. Kilka innych polimorfizmów było bliskich osiągnięcia znamienności statystycznej lub nie potwierdzono ich znaczenia przez różne grupy badawcze [59]. Ostatecznie w badaniach przeprowadzonych na ponad 250 tysiącach osób potwierdzono znaczenie wcześniej odkrytych 12 loci oraz odkryto kolejnych 18 loci w genomie związanych z wartościami BMI [60]. Ostatnie doniesienia zwiększyły liczbę loci w genomie człowieka wpływających na masę ciała do 97 [61].

Wyniki tych badań nie doprowadziły do wykrycia pojedynczego genu bezpośrednio wywołującego otyłość u człowieka, lecz raczej licznych zmian o niewielkim sumującym się działaniu. Wiele z nich wykazuje działanie plejotropowe, zwiększając ryzyko innych chorób, w tym cukrzycy, zaburzeń lipidowych. Szacuje się, że wpływ odkrytych licznych loci na BMI może odpowiadać ponad 20% zmienności [61]. Dalsze obserwacje wskazują na znacznie bardziej skomplikowane uwarunkowania w zakresie etiopatogenezy otyłości. Udowodniono, że działanie zmutowanego genu FTO jest wyraźnie modyfikowane przez aktywność fizyczną, która może zmniejszyć jego wpływ na BMI nawet o 30% [62]. Gen receptora aktywowanego proliferatorami peroksysomów typu γ (PPARγ) odgrywa istotną rolę w adipogenezie i metabolizmie lipidów. Wariant Pro12Ala tego genu zmniejsza wpływ bogatotłuszczowej diety stężenie lipidów w surowicy krwi oraz wzrost BMI w porównaniu do osób nie będących jego nosicielami [63]. Interesujące wyniki przyniosły badania na myszach poddanych eksperymentalnemu stłuszczeniu wątroby. Nieprawidłowe działanie białek regulujących odpowiedź immunologiczną takich jak TLR4 i TLR9, zmienia skład flory bakteryjnej jelit i prowadzi do rozwoju otyłości [64]. Z kolei w maju 2017 roku opublikowano wyniki metaanalizy GWAS przeprowadzonej u ponad 339 tysięcy osób z uwzględnieniem statystycznych wyników metaanalizy BMI (na podstawie 125 prac przeprowadzonych przez Genetic Investigation of ANthropometric Traits (GIANT) consortium). Celem pracy było lepsze zrozumienie biologicznych mechanizmów odpowiedzialnych za ryzyko rozwoju otyłości oraz odkrycie najważniejszych genów kandydatów poprzez powiązanie określonych loci w genomie z transkryptomem w tkance tłuszczowej (badanie poziomu ekspresji genów – liczba ich transkryptomów-cząsteczek mRNA, mikroRNA) oraz badaniami dotyczącymi metabolizmu adipocytów. Wyniki tych analiz wyłoniły gen GPD1L (zmapowany na chromosomie 3p22.3) jako najpoważniejszy gen kandydat w etiologii otyłości i insulinooporności. Ekspresja genu GPD1L wzrasta w podczas utraty i utrzymywania wagi spowodowanej dietą niskokaloryczną oraz obniża się w czasie przyrostu masy ciała wywołanego dietą wysokotłuszczową. Odkrycie to może mieć potencjalne znaczenie w poszukiwaniu terapeutycznej cząsteczki w leczeniu otyłości [65].

Cytowane powyżej badania odzwierciedlają zakres złożonej i bardzo trudnej problematyki związanej z poszukiwaniem związku przyczynowego pomiędzy identyfikowanymi w badaniach GWAS tysiącami wariantów w różnych loci w części kodującej genomu, których efekt fenotypowy w odniesieniu do ryzyka otyłości pozostaje często niepewny. Nie można wykluczyć, że tzw. odziedziczalność otyłości jest wyolbrzymiona, ponieważ nie można ściśle oddzielić znaczenia licznych czynników modyfikujących, w tym czynników środowiskowych działających na organizm już w trakcie życia płodowego (np. dieta wysokotłuszczowa u matki w trakcie trwania ciąży może implikować wystąpienie cech zespołu metabolicznego u potomstwa) jak również wpływ czynników epigenetycznych, które mogą być badanie z zastosowaniem nowych technik diagnostycznych jakimi są specyficzne mikromacierze genomowe umożliwiające określenie metylacji DNA określonych regionów w genomie. Potwierdzają to wieloletnie badania epidemiologiczne, kliniczne i na zwierzętach doświadczalnych wskazujące na rolę stresu oksydacyjnego w patogenezie otyłości poprzez zwiększenie proliferacji preadipocytów. Otyłość sama w sobie może powodować ogólnoustrojowy stres oksydacyjny poprzez różne mechanizmy biochemiczne związane z fosforylacją oksydacyjną. Wśród czynników które przyczyniają się do stresu oksydacyjnego w otyłości wymienia się takie jak: hyperglikemia, podwyższone stężenia lipidów, niedobory minerałów i witamin, przewlekłe infekcje, hyperleptynemia, zwiększona aktywność mięśni dla zapobiegania wzrostu masy ciała. [66].

Przedstawione w pracy informacje nie wyczerpują szerokiego zagadnienia jakim jest otyłość i jej genetyczne aspekty. Rozwój nowoczesnych technologii w biologii molekularnej bioinformatyki, transkryptomiki, proteomiki, biochemii sprzyja w ostatniej dekadzie postępowi w wiedzy na temat różnych aspektów związanych z otyłością i jej poważnymi w skutkach następstwami zdrowotnymi. Wyniki dotychczasowych wielokierunkowych badań jednocześnie wskazują na potrzebę dalszego pogłębiania wiedzy w tej dziedzinie i ukierunkowują poszukiwanie skutecznej terapii farmakologicznej w przyszłości.
